# RBM39 Promotes Base Excision Repair to Facilitate the Progression of HCC by Stabilising OGG1 mRNA

**DOI:** 10.1111/cpr.70059

**Published:** 2025-05-13

**Authors:** Hongda An, Anliang Xia, Siyuan Liu, Dongjun Luo, Longpo Geng, Binghua Li, Beicheng Sun, Zhu Xu

**Affiliations:** ^1^ Department of Hepatobiliary Surgery, Nanjing Drum Tower Hospital, Chinese Academy of Medical Sciences & Peking Union Medical College Graduate School of Peking Union Medical College Nanjing China; ^2^ Department of Hepatobiliary Surgery The First Affiliated Hospital of Anhui Medical University Hefei China; ^3^ MOE Innovation Center for Basic Research in Tumor Immunotherapy Hefei China; ^4^ Anhui Province Key Laboratory of Tumor Immune Microenvironment and Immunotherapy Hefei China

**Keywords:** base excision repair, hepatocellular carcinoma, mRNA stability, OGG1, RBM39

## Abstract

Targeting base excision repair (BER) has been an attractive strategy in cancer therapeutics. RNA‐binding motif protein 39 (RBM39) modulates the alternative splicing of numerous genes involved in cancer occurrence and progression. However, whether and how RBM39 regulates BER in hepatocellular carcinoma (HCC) remain unclear. Here, we found that under oxidative stress, RBM39 degradation or knockdown decreased BER efficiency in HCC cells using a well‐designed BER reporter. Further assays showed that RBM39 promoted HCC cell proliferation, migration, and invasion, enhancing cell survival and inhibiting apoptosis. Mechanistically, RBM39 interacted with the mRNA of the essential glycosidase 8‐oxoguanine‐DNA glycosylase 1 (OGG1), thereby stabilising OGG1 mRNA. This in turn increases OGG1 expression and promotes BER efficiency in HCC. Moreover, data suggested that RBM39 degradation, combined with oxidative damage, could be more effective for HCC treatment than monotherapy, both in vitro and in xenograft mice models. Overall, we demonstrated that RBM39 regulated OGG1 stabilisation and improved BER efficiency, suggesting that combining the RBM39 degradant indisulam with the oxidising agent KBrO_3_ could be an emerging strategy for HCC treatment.

## Graphical abstract

Targeting RBM39 attenuates BER via destabilising OGG1 mRNA and potentiates KBrO_3_‐induced oxidative stress in HCC. Our study represents a valuable treatment strategy combining indisulam and an oxidative stress inducer in HCC.

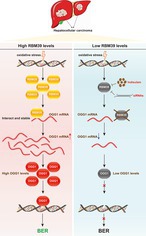

## 1 Introduction

Primary liver cancer constitutes a major global health burden as a leading cause of cancer‐related mortality worldwide, ranking third in incidence and second in mortality amongst digestive system malignancies according to the 2020 global cancer statistics [1]. Hepatocellular carcinoma (HCC), the predominant subtype of primary liver cancer, accounts for approximately 90% of cases and has a rapid increase in incidence over the past two decades [2, 3, 4, 5], emphasising HCC as a significant global health concern. Due to its high malignancy and insidious onset, HCC is often diagnosed at a middle to advanced stage [6, 7, 8, 9]. The recurrence rate in postoperative patients with advanced or metastatic HCC exceeds 70%, and the 5‐year survival rate is below 10% [10]. Thus, an in‐depth exploration of HCC mechanisms and novel treatments is essential to address this urgent need.

Studies have shown that genomic instability is a hallmark of human cancer, arising from DNA damage caused by various endogenous or exogenous sources [11, 12]. To respond to different types of DNA damage, the body relies on a robust repair system to inhibit tumorigenesis. Amongst the various DNA‐repair pathways, base excision repair (BER) is a highly conserved mechanism, from *E. coli* to humans, that repairs alkylated, deaminated and oxidised base lesions, apurinic/apyrimidinic (AP) sites, and single‐strand DNA breaks [13, 14, 15]. It has been estimated that each human cell needs to repair 10,000–20,000 DNA lesions per day [16]. Amongst these, 8‐oxoguanine (8‐oxoG) is one of the most studied BER lesions, inducible by potassium bromate (KBrO_3_) and abundant in DNA, with potential mutagenic properties [17]. Therefore, efficient BER is essential for maintaining genome integrity.

The BER pathway begins with the recognition and removal of damaged bases by DNA glycosylases, such as 8‐oxoguanine‐DNA glycosylase 1 (OGG1) [18]. The resulting abasic sites are further incised by AP endonuclease 1 (APE1) in coordination with poly(ADP‐ribose) polymerase 1 (PARP1), generating DNA nicks or single‐strand breaks (SSBs) with 5′‐deoxyribose phosphate (dRP). DNA Pol β then performs end processing of SSBs, filling gaps by incorporating the correct nucleotide and removing the 5′‐dRP. This is followed by the ligation of the SSB by X‐ray repair cross‐complementing protein 1 (XRCC1) in complex with DNA ligase IIIα (Lig3) to complete BER [12, 14, 19]. Recent studies indicate that individuals with impaired BER or abnormal BER protein expression have an increased risk of cancer. APE1 has been closely linked to the development and prognosis of colorectal cancer (CRC), prostate cancer, non‐small cell lung cancer (NSCLC), ER‐positive breast cancer, osteosarcoma, thyroid cancer and epithelial ovarian cancer [20]. Low Pol β expression correlates strongly with poor pathology and low survival rates in breast cancer, and Pol β mutations are primarily associated with CRC [12, 21]. XRCC1 variants R194W and R399Q increase the incidence of breast and pancreatic cancers [22, 23]. Furthermore, simultaneous knockout of OGG1/MYH raises the incidence of lung cancer, ovarian cancer and lymphomas in mice [24]. Despite these findings, the role of the BER pathway in HCC progression remains largely unknown.

RNA‐binding motif protein 39 (RBM39), an evolutionarily conserved U2AF paralog, emerges as a multimodal regulator integrating transcriptional coregulation, RNA splicing and immune response [25, 26]. As an important serine/arginine‐rich (RS) RNA‐binding protein, RBM39 is evolutionarily conserved in animals [27]. It contains one N‐terminal RS domain, two canonical RNA recognition motif (RRM) domains (RRM1 and RRM2), and one non‐canonical RRM domain (RRM3) at the C‐terminal [25]. The relationship between RBM39 and tumours is well established. Higher RBM39 expression correlates with worse neoplasm stages in colorectal cancer [28]. Depletion of RBM39 leads to large‐scale splicing defects in cancer, potentially disrupting multiple transcriptionally active pathways simultaneously [25]. RBM39 elimination by siRNA or CRISPR editing has shown a strong antiproliferative effect on various cancers, including acute myeloid leukaemia (AML), cholangiocarcinoma, CRC and breast cancer [29, 30, 31, 32]. Notably, RBM39 was initially identified in 1993 as an autoantigen in the serum of an HCC patient [33]. Recent studies have indicated that RBM39 regulates the transcription of metabolic genes in an arginine‐bound manner, thereby promoting hepatocarcinogenesis [34]. However, its direct role in oxidative stress‐mediated DNA damage repair remains unknown, and the underlying regulatory mechanism has not been elucidated. Indisulam, an aryl sulfonamide with selective anticancer activity, targets RBM39 for polyubiquitination and proteasomal degradation by recruiting it to the CUL4‐DCAF15 E3 ubiquitin ligase [31]. Previous research has demonstrated that indisulam‐induced degradation of RBM39 effectively inhibits pro‐oncogenic metabolic alterations in HCC, which highlights RBM39 as a promising candidate for HCC therapy development [34].

Here, our investigation reveals that RBM39 critically regulates the BER mechanism in HCC, identifying this RNA‐binding protein as a key modulator of DNA damage response pathways in liver cancer cells. In response to oxidative stress (OS), RBM39 protects tumour cells and inhibits apoptosis. Mechanistically, RBM39 interacts with OGG1 mRNA to stabilise OGG1 and promote the BER pathway. Consequently, RBM39 stimulates BER activity in HCC cells, both in vitro and in xenograft mouse models. Our study identified that RBM39 promotes HCC progression by transcriptionally activating OGG1, suggesting that combining indisulam with an oxidative damage inducer could be a promising therapeutic strategy for HCC.

## 2 Results

### 2.1 RMB39 Promotes BER Efficiency in HCC

**FIGURE 1:**
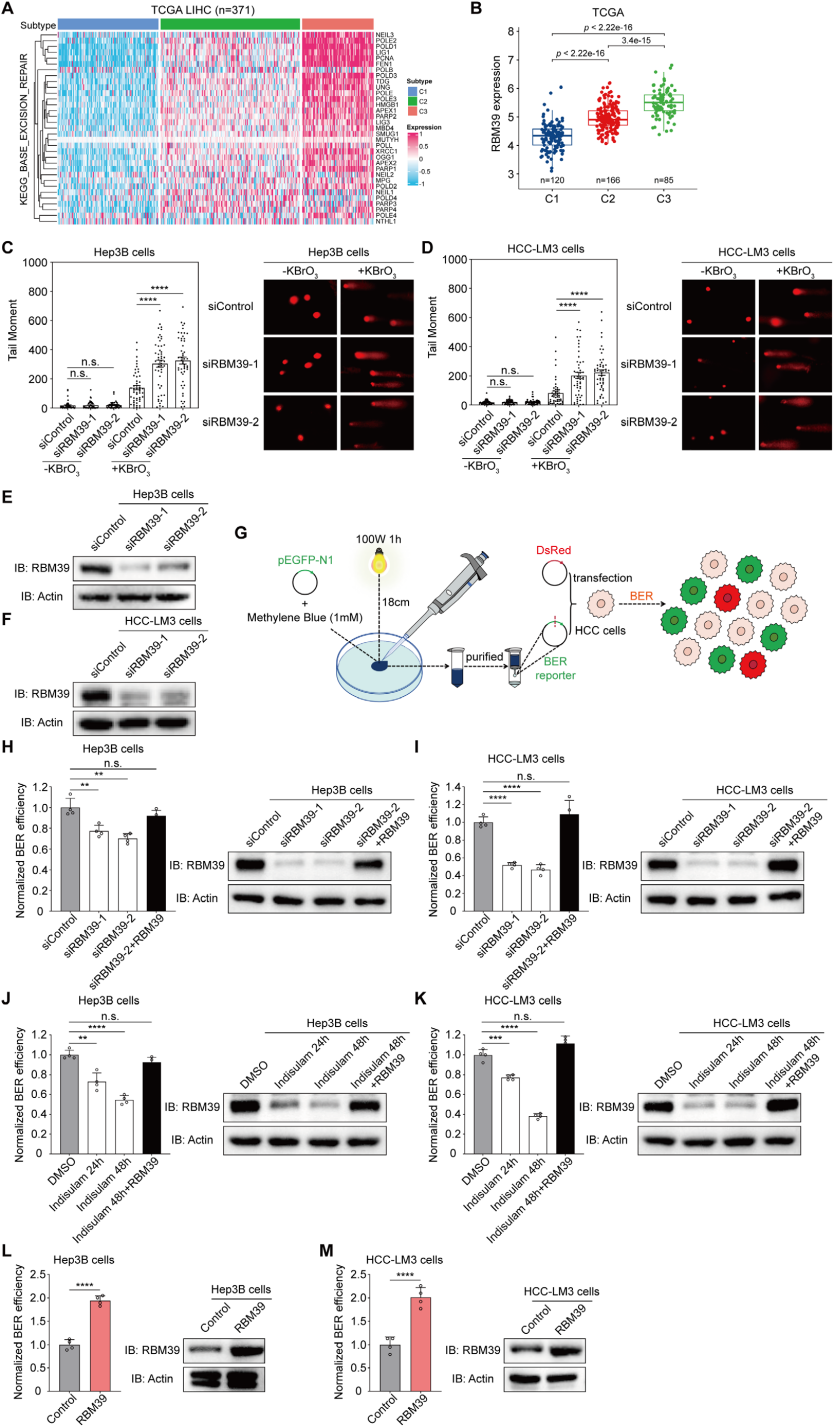
RBM39 promotes BER for maintaining genome stability. (A) The unsupervised consensus clustering based on the expression pattern of the 35 BER genes in HCC in TCGA database. (B) The expression of RBM39 in C1, C2 and C3 subtypes based on TCGA database. The analysis of genomic instability in control group and RBM39‐depleted Hep3B (C) and HCC‐LM3 (D) cells in the presence of the OS inducer KBrO_3_ using the alkaline comet assay and their representative images. The tail moment was considered as the measure of genomic instability, and at least 50 cells were analysed using the Comet Assay software Project. The Mann–Whitney *U* test was used for statistical analysis and the error bars indicated the S.E.M. values. Western blot analysis of RBM39 expression in control group and RBM39‐depleted Hep3B (E) and HCC‐LM3 (F) cells transfected with designated siRNAs. (G) Schematic diagram of the BER efficiency measurement. Analysis of BER efficiency and western blot in control group and RBM39‐depleted Hep3B (H) and HCC‐LM3 (I) cells transfected with designated siRNAs, part of the cells complemented 1 μg RBM39. Student's *t* test was used for statistical analysis and the error bars indicated the S.D. values. Analysis of BER efficiency and western blot in Hep3B (J) and HCC‐LM3 (K) cells treated with DMSO or indisulam, part of the cells complemented 1 μg RBM39. Student's *t* test was used for statistical analysis and the error bars indicated the S.D. values. Analysis of BER efficiency and western blot in control group and RBM39‐overexpressed Hep3B (L) and HCC‐LM3 (M) cells. Student's *t* test was used for statistical analysis and the error bars indicated the S.D. values. **p < 0.01; ***p < 0.001; ****p < 0.0001; n.s., not significant.

To identify whether RBM39 regulates the BER pathway in HCC, we first performed unsupervised consensus clustering using expression profiles of 35 BER‐related genes (Kyoto Encyclopedia of Genes and Genomes, KEGG). The results showed that patients with HCC in The Cancer Genome Atlas (TCGA) data set could primarily cluster into 3 subgroups, which we termed the C1, C2 and C3 subtypes, which represented low, intermediate and high expression levels of BER genes, respectively (Figures 1A and S1A). The prognostic analysis showed that the C3 subtype exhibited the shortest overall survival and progression‐free survival (Figure S1B,C). Unexpectedly, we found that C3 had the highest expression of RBM39 compared to the C1 and C2 subtypes, which indicated that RBM39 might have a stimulative role in BER (Figure 1B). To further confirm the effect of RBM39 in BER, we conducted a comet assay to analyse DNA damage in Hep3B and HCC‐LM3 cells with RBM39 knockdown and their corresponding controls exposed to OS induced by KBrO_3_. We observed that the tail moment, reflecting the severity of DNA damage, was significantly increased in Hep3B and HCC‐LM3 cells with RBM39 knockdown (Figure 1C–F). In contrast, under normal conditions, RBM39 knockdown had minimal or no impact on DNA damage. These data suggest that RBM39 is involved in repairing OS‐induced DNA damage.

To further verify this, we quantified BER efficiency in Hep3B and HCC‐LM3 cells using a well‐established fluorescence‐based plasmid reactivation assay [13, 19, 35]. The pEGFP‐N1 plasmid, which was treated with methylene blue and white light to induce 8‐oxodGuo damage, along with DsRed normalisation plasmids, was co‐transfected into cells (Figure 1G). Repair efficiency was calculated as the GFP+/DsRed+ fluorescence ratio via flow cytometry.

We observed that the BER efficiency in Hep3B and HCC‐LM3 cells with RBM39 knockdown by using siRNA (Figures 1H,I and S1D,E) or degradation by using indisulam (Figures 1J,K and S1F,G) was significantly reduced compared to controls. Consistently, the BER efficiency in RBM39‐overexpressed Hep3B (Figures 1L and S1H) and HCC‐LM3 (Figures 1M and S1I) cells was approximately 2‐fold that of normal cells. BER efficiency decreased more markedly when indisulam was applied for 48 h compared to 24 h. Furthermore, restoring RBM39 in RBM39 knockdown or degradation cells rescued BER efficiency. Taken together, RBM39 participates in KBrO_3_‐induced BER, enhancing BER efficiency to maintain genomic stability.

### 2.2 RBM39 Enhances Cell Survival and Inhibits Apoptosis in HCC

**FIGURE 2:**
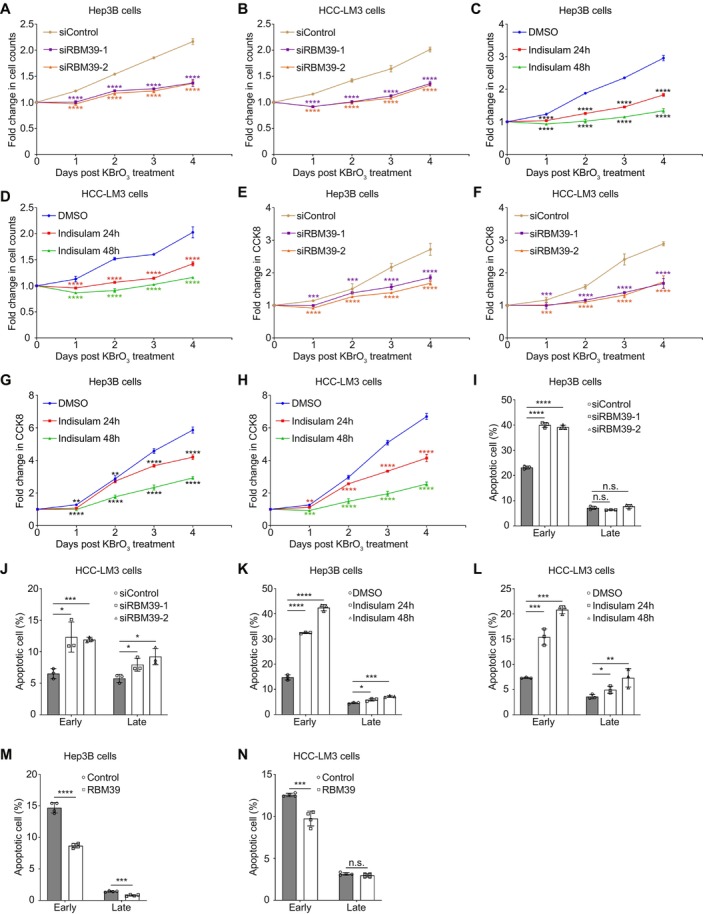
RBM39 improves cell proliferation and inhibits apoptosis. Cell count in control group and RBM39‐depleted Hep3B (A) and HCC‐LM3 (B) cells transfected with designated siRNAs. Cell count in Hep3B (C) and HCC‐LM3 (D) cells treated with DMSO or indisulam. Analysis of cell proliferation in control group and RBM39‐depleted Hep3B (E) and HCC‐LM3 (F) cells transfected with designated siRNAs. Analysis of cell proliferation in Hep3B (G) and HCC‐LM3 (H) cells treated with DMSO or indisulam. The analysis of apoptosis rates in control group and RBM39‐depleted Hep3B (I) and HCC‐LM3 (J) cells transfected with designated siRNAs. The analysis of apoptosis rates in Hep3B (K) and HCC‐LM3 (L) cells treated with DMSO or indisulam. The analysis of apoptosis rates in control group and RBM39‐overexpressed Hep3B (M) and HCC‐LM3 (N) cells. Student's *t* test was used for statistical analysis and the error bars indicated the S.D. values. *p < 0.05; **p < 0.01; ***p < 0.001; ****p < 0.0001; n.s., not significant.

To assess whether RBM39 influences cell survival by enhancing BER efficiency, we evaluated how RBM39 affects cell proliferation under OS. We found that RBM39 knockdown (Figures 2A,B and S2A) or degradation (Figures 2C,D and S2B) significantly reduced cell counts in Hep3B and HCC‐LM3 cells exposed to KBrO_3_ or H_2_O_2_. These results were confirmed by a CCK8 assay (Figures 2E–H and S2C,D) that showed RBM39 knockdown or degradation decreased proliferation rates. Both survival and proliferation rates declined further with extended indisulam treatment.

To further explore the relationship between RBM39 and cell proliferation, we examined how RBM39 affects apoptosis under OS conditions. We observed that siRNA‐mediated RBM39 knockdown increased KBrO_3_‐induced early apoptosis by approximately 2‐fold (Figures 2I,J and S2E,F). Indisulam‐mediated RBM39 degradation for 24 or 48 h increased early apoptosis by about 2‐fold or 3‐fold, respectively (Figures 2K,L and S2G,H). Late apoptosis induced by both methods was slightly or not significantly affected. Additionally, overexpressing RBM39 noticeably reduced KBrO_3_‐induced early apoptosis in Hep3B (Figures 2M and S2I) and HCC‐LM3 (Figures 2N and S2J) cells. Together, these findings suggest that RBM39 enhances cell proliferation and primarily inhibits early apoptosis by increasing BER efficiency.

### 2.3 RBM39 Promotes the Expression of OGG1 in HCC

**FIGURE 3:**
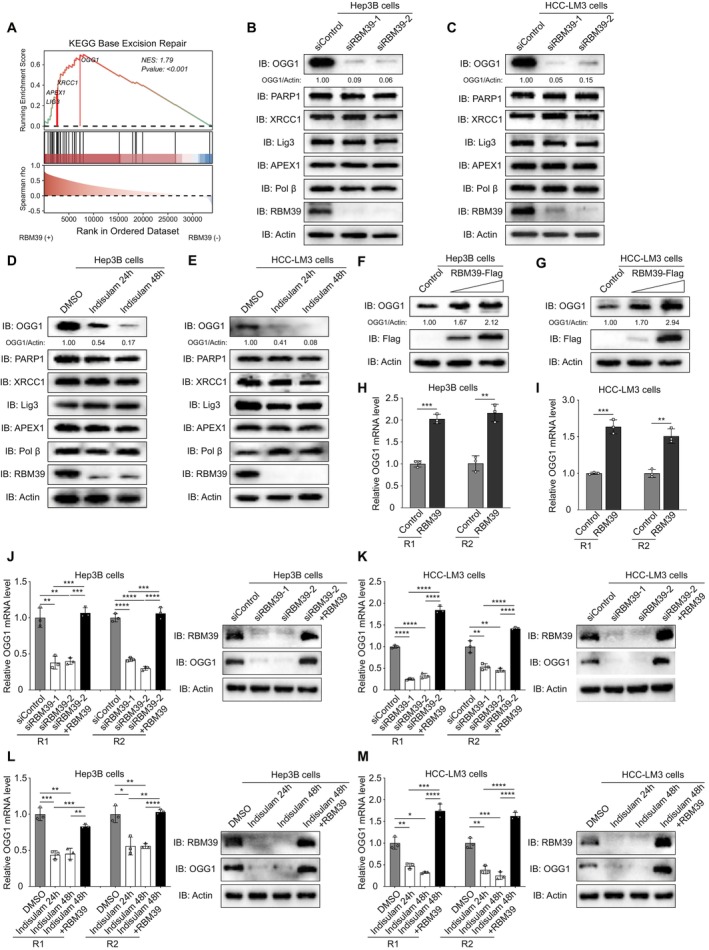
RBM39 regulates the expression of OGG1 protein and mRNA. (A) The correlations between RBM39 and BER‐related genes based on Kyoto Encyclopedia of Genes and Genomes database. Western blot analysis of BER pathway‐related protein expression in Hep3B (B) and HCC‐LM3 (C) cells transfected with designated siRNAs. Western blot analysis of BER pathway‐related protein expression in Hep3B (D) and HCC‐LM3 (E) cells treated with DMSO or indisulam. Western blot analysis of OGG1 protein levels in control group and RBM39‐overexpressed Hep3B (F) and HCC‐LM3 (G) cells. Analysis of OGG1 mRNA levels in control group and RBM39‐overexpressed Hep3B (H) and HCC‐LM3 (I) cells. (J–M) RBM39 rescued the OGG1 mRNA levels in RBM39‐depleted Hep3B and HCC‐LM3 cells. **p < 0.01; ***p < 0.001 ****p < 0.0001.

To investigate how RBM39 regulates the BER pathway in HCC, we grouped RBM39 and BER factors based on the TCGA database. Results showed that RBM39 was associated with the expression of multiple key proteins in the BER pathway (Figure 3A). To further determine which BER factors were regulated by RBM39, we tested the expression of key BER factors in Hep3B and HCC‐LM3 cells with RBM39 knockdown or degradation. We found that RBM39 knockdown (Figure 3B,C) or degradation (Figure 3D,E) significantly reduced the protein level of N‐glycosylase OGG1, whilst other essential BER factors showed no significant changes. Consistent with this, overexpressing RBM39 also substantially increased OGG1 protein levels in a dose‐dependent manner (Figure 3F,G). Additionally, the presence of KBrO_3_ further elevated OGG1 protein levels in RBM39‐overexpressing Hep3B (Figure S3A) and HCC‐LM3 (Figure S3B) cells.

Given OGG1's protein level, we examined OGG1 mRNA levels to determine whether RBM39 regulates its mRNA level. RT‐qPCR results showed that RBM39 overexpression in Hep3B (Figure 3H) and HCC‐LM3 (Figure 3I) cells significantly increased OGG1 mRNA levels by about 2‐fold. Furthermore, RBM39 knockdown (Figure 3J,K) or degradation (Figure 3L,M) significantly decreased OGG1 mRNA levels, whilst RBM39 re‐overexpression in RBM39‐depleted Hep3B (Figure 3J,L) and HCC‐LM3 (Figure 3K,M) cells significantly rescued OGG1 mRNA levels, suggesting that RBM39 regulates OGG1 expression through regulating the mRNA level of OGG1. In summary, similar trends observed in protein and mRNA levels confirm that RBM39 regulates OGG1 mRNA and plays a critical role in promoting the BER pathway.

### 2.4 RBM39 Binds to the mRNA of OGG1 and Plays an Important Role in HCC

**FIGURE 4:**
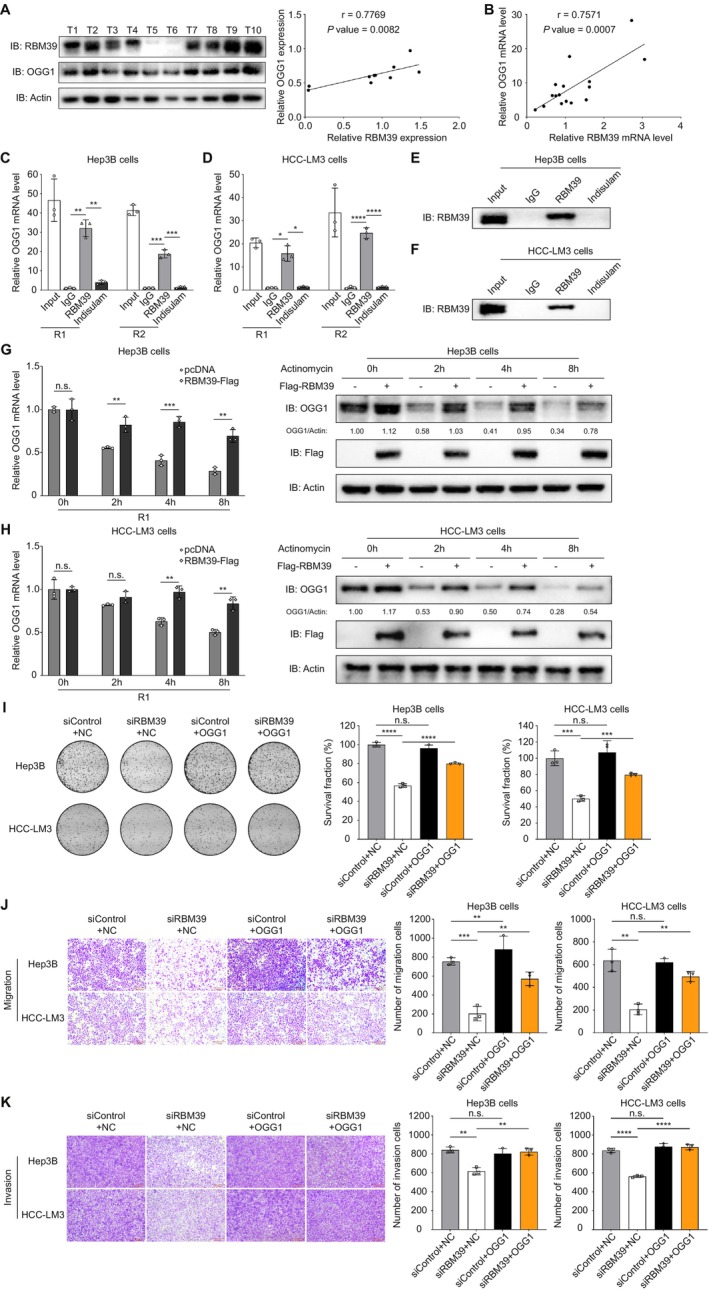
RBM39 interacts with OGG1 mRNA, and OGG1 mediates RBM39‐induced progression of HCC. (A) The protein expression levels of RBM39 and OGG1 in tumour tissues of 10 HCC patients were compared by WB method. (B) The mRNA levels of RBM39 and OGG1 in tumour tissues of 16 HCC patients were compared by RT‐qPCR method. RIP assays showed the interaction of RBM39 with OGG1 mRNA in Hep3B (C) and HCC‐LM3 (D) cells. Western blotting assays of the specific interaction of RBM39 with OGG1 mRNA in Hep3B (E) and HCC‐LM3 (F) cells. The Hep3B (G) and HCC‐LM3 (H) cells in control group and RBM39‐overexpressed group were treated with actinomycin at 5 μg/mL for the indicated times. Samples were collected for quantitative RT‐PCR and western blot analysis to detect the expression level of OGG1. The R1‐F1 primers of OGG1 were used in quantitative RT‐PCR. (I) Overexpression of OGG1 rescued the colony formation ability of Hep3B and HCC‐LM3 cells with RBM39 knockdown. (J) Overexpression of OGG1 rescued the migration ability of Hep3B and HCC‐LM3 cells with RBM39 knockdown. (K) Overexpression of OGG1 rescued the invasion ability of Hep3B and HCC‐LM3 cells with RBM39 knockdown. *p < 0.05; **p < 0.01; ***p < 0.001; ****p < 0.0001; n.s., not significant.

To explore the potential involvement of RBM39 and OGG1 in HCC, we conducted a comparative analysis of their transcriptional profiles between tumour and matched peri‐tumoral tissues using the GEPIA database. Results showed that RBM39 (Figure S4A) and OGG1 (Figure S4B) expression levels were both significantly upregulated in tumour tissues compared to adjacent non‐tumour tissues. Interestingly, we found that RBM39 had a strong positive correlation with OGG1 in multiple human cancers based on the TCGA database and the GEPIA database (Figure S4C,D). These data were consistent with our western blot (Figure 4A) and RT‐qPCR (Figure 4B) results by using clinical HCC samples, suggesting that RBM39 might bind to OGG1 mRNA to regulate its stability.

To further clarify the molecular mechanism by which RBM39 affects OGG1 in cells, we performed an RNA immunoprecipitation (RIP) assay. Results showed that OGG1 mRNA specifically bound to RBM39 in Hep3B cells and HCC‐LM3 cells (Figure 4C–F). However, OGG1 mRNA binding to RBM39 was absent in cells treated with indisulam in the RIP assay (Figure 4C–F). Using the catRAPID database (http://s.tartaglialab.com/page/catrapid_group), we predicted the possible binding sites, identifying amino acids 301–352 of RBM39 and nucleotides 262–321 of OGG1 as the most likely binding regions (Figure S4E).

To determine whether RBM39 regulates OGG1 mRNA stability, cells were treated with the transcription inhibitor actinomycin D (5 μg/mL) for specific time intervals. We found that OGG1 mRNA levels gradually declined in actinomycin D‐treated control cells, whereas RBM39 overexpression delayed OGG1 mRNA decay in Hep3B (Figures 4G and S4F) and HCC‐LM3 (Figures 4H and S4G) cells, indicating that RBM39 stabilises OGG1 transcripts. Western blot analysis showed that actinomycin D inhibited OGG1 protein expression in Hep3B (Figure 4G) and HCC‐LM3 (Figure 4H) cells, whilst RBM39 overexpression partially restored OGG1 expression, further suggesting that RBM39 stabilises OGG1 mRNA. Moreover, the inhibition of proliferation (Figure 4I), migration (Figure 4J), invasion (Figure 4K) and wound healing (Figure S4H,I) observed in RBM39 knockdown Hep3B and HCC‐LM3 cells was reversed by OGG1 re‐overexpression. Collectively, these results demonstrate that RBM39 interacts with OGG1 mRNA, promoting HCC progression by regulating the BER pathway.

### 2.5 Combination of RBM39 Degradant and KBrO_3_ Is a Potential Strategy for the Treatment of HCC

**FIGURE 5:**
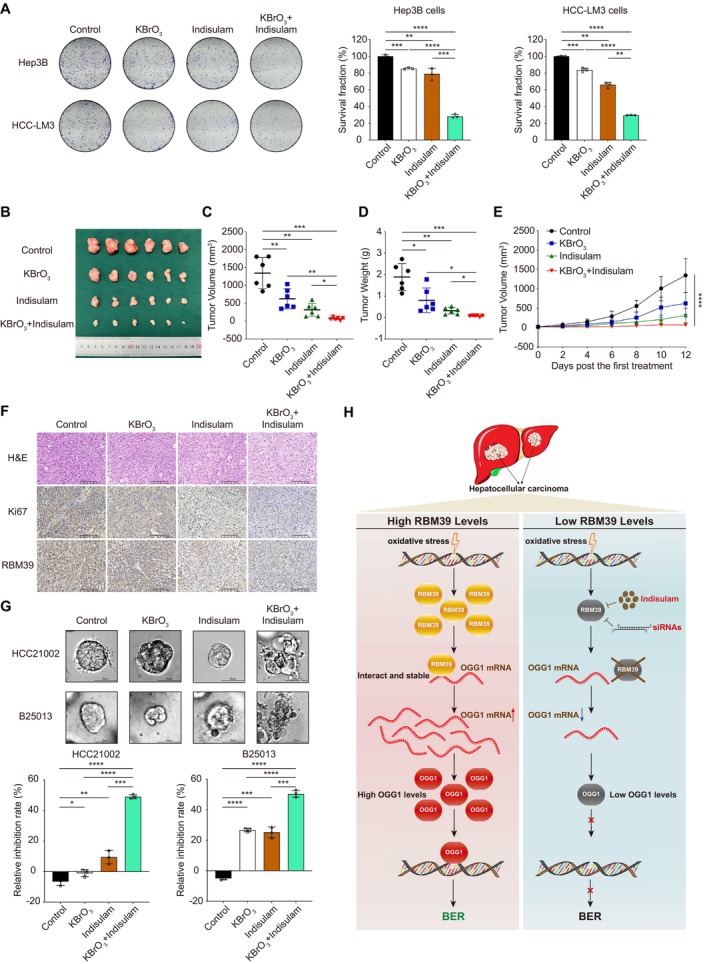
Combination of KBrO_3_ and indisulam synergistically inhibits HCC growth. (A) Colony formation images and colony count results of the Hep3B and HCC‐LM3 cells treated with DMSO, 20 mM KBrO_3_, 1 μM indisulam or 20 mM KBrO_3_ combined with 1 μM indisulam. Tumour image (B), volume (C), weight (D) and volumetric change (E) of tumours harvested in nude mice subcutaneously injected with Hep3B cells. Mice were intraperitoneally injected with control solution, 20 mg/kg KBrO_3_, 25 mg/kg indisulam or 20 mg/kg KBrO_3_ combined with 25 mg/kg indisulam once a day for 12 days. (F) Representative H&E and immunohistochemistry images of the expression of RBM39 and Ki67 in the xenograft tumours in each group. Scale bar, 100 μm. (G) The images and relative inhibition rate results of the HCC21002 and B25013 HCC organoids treated with DMSO, 370 μM KBrO_3_, 1 μM indisulam or 370 μM KBrO_3_ combined with 1 μM indisulam. Scale bar, 50 μm. *p < 0.05; **p < 0.01; ***p < 0.001; ****p < 0.0001. (H) A proposed working model of RBM39 in BER pathway.

Given the important role of RBM39 in the BER pathway, we sought to determine whether a combination of RBM39 degradant and KBrO_3_ could effectively inhibit HCC. Colony formation assays showed that KBrO_3_ or indisulam alone reduced the number of colonies in multiple HCC cells compared to controls, whilst the combination exhibited greater inhibition effects, with a significant reduction in colony numbers compared to either treatment alone (Figures 5A and S5A). This evidence suggests that the combined use of KBrO_3_ and indisulam effectively inhibits HCC cell proliferation.

To confirm these findings, we established a subcutaneous xenograft model of HCC in BALB/c mice. In Hep3B subcutaneous tumours, the combination of KBrO_3_ and indisulam significantly reduced tumour volume (Figure 5B,C) and weight (Figure 5D) compared to the control, KBrO_3_ and indisulam groups. Dynamic tumour volume measurements revealed that the combination therapy inhibited tumour growth and maintained low proliferation levels (Figure 5E). Immunohistochemistry analysis confirmed varying degrees of proliferation activity and RBM39 expression across the groups (Figure 5F). Moreover, HCC organoid assays showed that KBrO_3_ or Indisulam alone reduced the relative inhibition rates of HCC21002 and B25013 organoids compared to controls, whilst the combination exhibited greater inhibition effects, with a significant reduction in relative inhibition rates compared to either treatment alone (Figures 5G and S5B). Overall, the combination of KBrO_3_ and indisulam in Hep3B and HCC‐LM3 cells demonstrated synergistic effects, suggesting that this strategy could effectively inhibit HCC cell proliferation.

Based on these findings, we deduced that RBM39 participated in the BER pathway by stabilising the mRNA of OGG1 (Figure 5H). Taken together, these results highlighted the importance of RBM39 for OS‐mediated DNA damage repair in HCC, and the combination of KBrO_3_ and indisulam might be a new strategy for HCC treatment (Figure 5H).

## 3 Discussion

According to the World Health Organisation, HCC is the fifth most common malignancy globally and a leading cause of cancer‐related death in the United States. Whilst advances in surgical resection, radiofrequency ablation, interventional techniques and liver transplantation have improved outcomes for early‐stage HCC [2, 36], effective strategies for advanced HCC remain urgently needed. RBPs play an important role in tumorigenesis and the development of many cancers, and targeting RBPs has vital potential for cancer therapeutics. RBM39, a pre‐mRNA splicing factor and transcription coactivator, is overexpressed in the majority of human cancers. Its role in tumorigenesis and tumour immunity suggests that RBM39 could serve as a biomarker for tumorigenesis and treatment [27, 37].

BER is responsible for mending the majority of oxidative, alkylative and hydrolytic DNA damage generated by both endogenous and exogenous sources. Alterations in BER capacity or the levels of BER proteins could significantly influence cancer progression and therapy. Based on the expression pattern of the 35 BER genes, HCC could primarily cluster into C1, C2 and C3 subgroups, indicating that targeting BER would be a promising strategy for HCC treatment. Our data revealed the molecular mechanism by which RBM39 influences BER efficiency in HCC. Under OS, RBM39 stabilised OGG1 by interacting with OGG1 mRNA, thereby promoting the BER pathway and facilitating HCC cell proliferation, migration and metastasis. The combination of the RBM39 degradant indisulam and the oxidising agent KBrO_3_ synergistically inhibited the growth of HCC cells both in vitro and in subcutaneous tumours in mice, presenting a promising treatment strategy for HCC.

A prior study demonstrated that RBM39 potentially plays a role in cellular defence against OS originating from endogenous metabolic processes, given that cells lacking RBM39 exhibited impaired antioxidant capacity and developed autophagy‐like phenotypes [38]. Our results revealed a novel mechanism in which RBM39 responds to OS by positively regulating OGG1 expression in the BER pathway. Given RBM39's potential role in DNA damage repair, we examined its expression in cells using an alkaline comet assay and a BER reporter to assess the effect of RBM39 on cell survival and apoptosis. We observed that RBM39 depletion significantly increased DNA damage in HCC cells under OS by reducing BER efficiency, thus promoting apoptosis and inhibiting HCC growth.

Several studies have shown that the interaction between RBM39 and RNA is closely related to tumour progression. RBM39 promotes myeloma cell survival under hypoxic conditions by binding to the long non‐coding RNA (lncRNA) DARS‐AS1, and the cancer‐testis lncRNA LINC01977 interacts with RBM39 to promote HCC progression by inhibiting Notch2 ubiquitination and degradation [39, 40]. Our study adds evidence that RBM39's interaction with OGG1 mRNA promotes HCC development. Consistent with a previous study showing that RBM39 knockdown affects HCC cells in CCK‐8 and wound healing assays [41], our findings confirmed that RBM39 knockdown inhibits HCC cell proliferation, migration and metastasis as shown by transwell assays. Additionally, we observed that OGG1 overexpression could significantly rescue the colony formation and membrane penetration ability of HCC cells with RBM39 knockdown, indicating that OGG1 is an essential downstream target of RBM39. Researchers have shown that RBM39's regulation of pre‐mRNA splicing influences necrocytosis in neuroblastoma, DNA damage repair in breast cancer and AML lethality [29, 42, 43]. Our investigation demonstrated that RBM39 transcriptionally regulates OGG1 expression, suggesting that RBM39 may stabilise OGG1 mRNA through pre‐mRNA splicing.

Guanine has the lowest redox potential amongst the five nucleobases, making it highly susceptible to oxidation, resulting in 8‐oxoG production in DNA or in the deoxynucleotide pool, which is primarily removed by OGG1 [44]. Consistent with previous findings that OGG1 overexpression stimulates HCC cell proliferation and enhances oxidative damage repair [45], our results confirmed that RBM39‐mediated OGG1 overexpression promotes HCC progression through the BER pathway. Evidence from a recent study suggests that upregulated OGG1 promotes synaptotagmin 7 transcription, leading to E‐cadherin consumption and metastasis in human lung adenocarcinoma [46]. Our data similarly indicated that OGG1 complementation enhanced HCC cell migration and metastasis, suggesting that OGG1 may also promote epithelial‐mesenchymal transition in tumours.

Indisulam, a carbonic anhydrase degradant, mediates RBM39 degradation by enhancing the interaction between DCAF15 and RBM39's RRM2 [47]. Interestingly, we observed that RBM39's RRM2 domain likely binds OGG1 mRNA, suggesting that indisulam‐induced RBM39 degradation might impair its ability to stabilise OGG1 mRNA. Research has shown that overactivation of YAP/TAZ axis is common in cancers, enabling resistance to indisulam and stabilising RBM39 [48, 49]. In our study, indisulam at 1 μM in cells and 25 mg/kg in mice effectively degraded RBM39. Numerous studies have reported that indisulam inhibits proliferation in various cancers, including gastric, cervical and neuroblastoma, as well as AML and T‐cell acute lymphoblastic leukaemia, by promoting RBM39 degradation [50, 51, 52, 53, 54, 55]. Our data indicated that indisulam also inhibits HCC cell proliferation in vitro and in subcutaneous xenografts in nude mice. Although indisulam has completed Phase I/II clinical trials both as monotherapy and in combination with chemotherapeutic agents [25], no complete remission has been observed in most patients, and no clinical trials have been reported for HCC.

To investigate whether indisulam affects HCC through the BER pathway, we found that whilst indisulam or KBrO_3_ alone could reduce tumour volume and weight to some extent, their combination produced a more significant synergistic anti‐HCC effect. Additionally, recent studies suggest that exosomal miR‐2682 and ubiquitin‐specific protease 39, as upstream regulators of RBM39, affect tumorigenesis in lung cancer and gastric cancer progression, respectively [56, 57]. However, finding drugs to precisely target these upstream regulators in clinical settings remains challenging. A previous report indicated that combining OGG1 and PARP1 inhibitors showed greater efficacy than monotherapy in BRCA1‐deficient breast cancer [58]. Compared to an OGG1 inhibitor, indisulam not only has specific tumour degradation mechanisms and advanced clinical trials but also indirectly inhibits OGG1 through RBM39. Our data add a critical piece to the puzzle regarding indisulam treatment.

In summary, this study demonstrates that RBM39 increases OS‐induced BER efficiency in HCC cells. RBM39 is highly expressed in HCC, accelerates HCC progression and inhibits apoptosis. Mechanistic studies reveal that RBM39 directly interacts with OGG1 mRNA, enhancing its stability. Therefore, RBM39 is a BER modulating protein and a potential cancer therapy target, suggesting that combining indisulam with KBrO_3_ offers a promising strategy to improve HCC treatment outcomes.

## 4 Materials and Methods

### 4.1 Clinical Specimens

The samples of fresh tissue were obtained from patients with HCC confirmed by postoperative pathological examination in Nanjing Drum Tower Hospital from 2020 to 2021. After surgical resection, all tissues were immediately placed in liquid nitrogen. This study was conducted according to the protocols approved by the Institutional Ethics Committee of Nanjing Drum Tower Hospital. All experimental procedures are in compliance with the Government's policies and the guidelines of the Declaration of Helsinki. Analysis and experiments were carried out with the understanding and written consent of each participant.

### 4.2 Cell Culture

All Hep3B, HCC‐LM3, MHCC‐97H and Huh‐7 cells were cultured in Dulbecco's modified Eagle's medium (Wisent corporation, Cat. # 319‐005‐CL) supplemented with 10% fetal bovine serum (Wisent corporation, Cat. # 087‐450) and 1% penicillin/streptomycin (Wisent corporation, Cat. # 450‐201‐EL), incubated in a humidified incubator containing 5% CO2 at 37°C. All cell lines were routinely monitored for mycoplasma and were negative.

### 4.3 Plasmid Transfection and Reagents

The ORF of RBM39 with a C‐terminal Flag tag was cloned into the pLVX vector backbone. The pEGFP‐N1 vector was modified to contain the OGG1 ORF fused to a His tag at its C‐terminus. The siRNAs were purchased from the Corues Biotechnology Company of Nanjing. The target sequences used in this study were as follows: RBM39‐siRNA‐1, 5′‐GGAACAACUUAAUGGAUUUTT‐3′; RBM39‐siRNA‐2, 5′‐GAUUAAGGAUGAUGUGAUUTT‐3′.

For over‐expressing RBM39 and OGG1, expression plasmid was transfected into Hep3B and HCC‐LM3 cells by using polyethyleneimine (PEI). For the detection of BER efficiency, the electrotransfection reagent SF Cell Line Solution Box (Lonza, Cat. # PBC2‐00675) was mixed with Hep3B or HCC‐LM3 cells and transferred into the 16‐well NucleocuvetteTM Strip (Lonza, Cat. # PDH‐2104), and transfected on Lonza 4D electroporator using DT‐130 program. All siRNAs was performed using ExFect Transfection Reagent (Vazyme, Cat. # T101‐01) following the manufacturer's instructions.

The chemical reagents used in the study included potassium bromate (KBrO_3_) (Adamas‐beta, Cat. # 82673); H_2_O_2_ (Jiangxi Caoshanhu, Cat. # JCSH001); Indisulam (MedChemExpress, Cat. # HY‐13650) and RNA synthesis inhibitor actinomycin D (MedChemExpress, Cat. # HY‐17559).

### 4.4 Alkaline Comet Assay

Hep3B and HCC‐LM3 cells were seeded at a density of 5 × 10^4^ cells/well in six‐well plates. On Day 2, cells were treated with KBrO_3_ at a concentration of 40 mM for 0.5 h. Following collection, cells were resuspended in PBS and adjusted to a density of 1 × 10^6^ cells/ml prior to conducting the comet assay. All experimental steps were carried out in strict adherence to the manufacturer's specifications (Beyotime Biotechnology, Cat. # C2041S) with comet assay slides (Beyotime Biotechnology, Cat. # FSL061‐5pcs) and coverslips (Beyotime Biotechnology, Cat. # FCGF18). Tail moments were used to quantitatively determine the amount of DNA damage using Comet Assay software Project (CASP1.2.3 beta 1).

### 4.5 Analysis of BER Efficiency

The pEGFP‐N1 plasmid was treated with 1 mM methylene blue and exposed to visible light irradiation (100 W) at an 18 cm distance for 1 h. The damaged pEGFP‐N1 vector was then purified by FastPure Gel DNA Extraction Mini Kit (Vazyme, Cat. # DC301‐01) and cotransfected with a vector encoding DsRed into cells. On Day 3 post transfection, cells were harvested for fluorescence‐activating cell sorter (FACS) analysis. The ratio of GFP+ cells to DsRed+ cells was used to represent the efficiency of BER.

### 4.6 Survival Assay, CCK‐8 Assay and FACS Analysis of Apoptosis

Hep3B and HCC‐LM3 cells were respectively seeded at a density of 1 × 10^5^ cells per plate. On Day 2 post‐seeding, cells were treated with 1 μM indisulam or transfected with RBM39‐siRNAs. On Day 3 post‐seeding, cells were treated with 20 mM KBrO_3_ for 1 h and then cultured in drug‐free medium for the indicated times. CCK‐8 assays were performed according to the instructions of the CCK‐8 kit (Vazyme, Cat. # A311‐01) and the cell absorptivity was measured at the wavelength of 450 nm by Multi‐Function Measuring Instrument (Tecan Spark, Switzerland). Cells were counted by Countstar BioLab (Shanghai RuiYu Biosciences, China) to measure the variation of cell numbers. Besides, cells were harvested according to the manufacturer's instructions for the apoptosis assay with an Annexin V‐FITC Apoptosis Detection Kit (Beyotime Biotechnology, Cat. # C1062S) for FACS analysis.

### 4.7 FACS Analysis

Cells were harvested and resuspended in 0.3 mL PBS for FACS analysis on a CYTEK Dxp Athena flow cytometer (CYTEK Biosciences, USA). At least 20,000 events were analysed. All data were subsequently analysed using FlowJo_V10.0.7 software.

### 4.8 Western Blotting

Protein extraction from cells or tissues was performed with RIPA buffer supplemented with protease and phosphatase inhibitors as previously described [59]. Sample protein concentrations were measured using a BCA assay kit (Vazyme, E112‐02), followed by loading 20 μg of protein per lane. Protein samples were electrophoresed on SDS‐PAGE gels and subsequently transferred onto PVDF membranes with 0.45 μm pore size (Millipore, USA). Following blocking with 5% skim milk in TBST for 1 h at room temperature, the membranes were probed with specific primary antibodies at 4°C overnight. After TBST washes the next day, secondary antibody incubation was performed for 1 h at room temperature, followed by additional TBST washes. Finally, the target protein bands were detected using SuperFemto ECL Chemiluminescence Kit (Vazyme, Cat. # E423‐01) and visualised in ChemiDoc XRS+ Chemiluminescence image system (Bio‐Rad Laboratories, USA). Antibodies against β‐actin (AC038), PARP1 (A0942), Lig3 (A1887) and OGG1 (A2268) were from ABclonal, whilst antibodies against RBM39 (21339‐1‐AP), XRCC1 (21468‐1‐AP), APEX1 (10203‐1‐AP), OGG1 (15125‐1‐AP) and Pol β (18003‐1‐AP) were from Proteintech.

### 4.9 Quantitative RT–PCR

Total RNA isolation was performed using a commercial extraction kit (Vazyme, Cat. # RC113‐C1), followed by cDNA synthesis with the HiScript III 1st Strand cDNA Synthesis Kit (Vazyme, Cat. # R312‐02) according to the manufacturer's protocol. Real‐time quantitative PCR was performed with ChamQ Universal SYBR qPCR Master Mix (Vazyme, Cat. # Q711‐03) on a ViiA 7 Real‐Time PCR system (Applied Biosystems). The mean Ct values from triplicate measurements were calculated, and relative expression was determined through ΔΔCt analysis. The relative expression levels were normalised to the level of Actin. The primers used to amplify OGG1 were as follows: OGG1‐mRNA‐F‐1, 5′‐CACACTGGAGTGGTGTACTAGC‐3′; OGG1‐mRNA‐R‐1, 5′‐CCAGGGTAACATCTAGCTGGAA‐3′; OGG1‐mRNA‐F‐2, 5′‐ACTCCCACTTCCAAGAGGTG‐3′; OGG1‐mRNA‐R‐2, 5′‐GGATGAGCCGAGGTCCAAAAG‐3′.

### 4.10 RNA Binding Protein Immunoprecipitation (RIP)

Firstly, sufficient cell lysate was obtained from Hep3B cells or Hep3B cells treated with 1 μM indisulam. Then the magnetic beads were incubated with IgG (Sigma, Cat. # 17‐701) and RBM39 (Proteintech, Cat. # 21339‐1‐AP) antibodies for 30 min at room temperature. The lysates were respectively incubated with the magnetic bead‐antibody complex by rotating at 4°C for 3 h to overnight. Proteinase K digestion and phenol‐chloroform/ethanol precipitation were conducted to isolate purified RNA after washing the beads a total of six times. Finally, qRT‐PCR was used to verify the expression of OGG1 and IgG.

### 4.11 Colony Formation Assays

A total of 1000 cells per well were seeded into six‐well plates for colony formation assays. Cells were transfected with RBM39‐siControls or RBM39‐siRNAs on Day 2 post‐seeding and overexpressed OGG1 on Day 3 post‐seeding for knocking down RBM39‐related colony formation assays. On Day 2 post‐seeding, cells were treated with DMSO, 20 mM KBrO_3_, 1 μM indisulam and 20 mM KBrO_3_ combined with 1 μM indisulam separately for HCC treatment‐related assays. Following approximately 9 days of culture, cells were fixed with 4% paraformaldehyde (Servicebio, Cat. #G1101‐500ML) for 30 min at room temperature, then subjected to crystal violet staining (Beyotime Biotechnology, Cat. # C0121‐100 mL) at 37°C for 30 min. Then, colonies containing more than 50 cells were photographed and counted.

### 4.12 Transwell Assay

For migration assay, a total of 100,000 cells per well were seeded into the upper chamber of tissue culture plate inserts (Biofil, Cat. # TCS003024). For invasion assay, a total of 200,000 cells per well were inoculated into the upper chamber of cell invasion chamber with 24‐well plates (LABSELECT, Cat. # 14347). After being cultured for 48 h, cells were fixed using 4% polyformaldehyde at room temperature for 30 min and stained with crystal violet staining at 37°C for 30 min.

### 4.13 Wound Healing Assay

A total of 600,000 cells per well were seeded in six‐well plates. After 24 h of siRNA transfection, once the cells reached 95% confluence, a straight scratch was made in the monolayer using a 200 μL pipette tip. Cells were maintained in serum‐free DMEM throughout the experiment. Migration was monitored by capturing images at 0, 24, 48 and 72 h under an inverted microscope (10× magnification).

### 4.14 Immunohistochemistry (IHC)

Following mounting of 8‐μm paraffin sections on slides, sequential dehydration–rehydration was performed using alcohol‐xylene solutions. Antigen epitopes were exposed with Tris‐EDTA (pH 9.0), followed by peroxidase inhibition and non‐specific binding blocking with serum at ambient temperature. The primary antibody RBM39 antibody (Proteintech, Cat. # 21339‐1‐AP) or Ki67 antibody (ABclonal, Cat. # A20018) was added to the tissues and incubated for 12 h at 4°C. Next, the slides were subjected to three PBST washes prior to 1 h incubation with secondary antibody at room temperature. Finally, the tissues were developed using DAB solution, stained with haematoxylin, dehydrated with ethanol, and sealed. Sample analysis and imaging were performed using light microscopy.

### 4.15 Animal Experiments

BALB/c male nude mice (4 weeks old) were obtained from GemPharmatech Co. Ltd. and housed under specific pathogen‐free conditions. All mice were housed at 20°C ± 2°C under a 12‐h light–dark cycle with lights on at 6:30 and provided with food and water AD libitum. All experimental protocols involving animals were conducted in accordance with the Guide for the Care and Use of Laboratory Animals. Mice were subcutaneously injected with Hep3B cells (5 × 10^6^ /mouse) on the right side and randomly divided into 4 groups, with 6 mice per group. Seven days after injection, therapy was started with vehicle control (10% DMSO, 40% PEG300, 5% Tween 80, 45% saline), 20 mg/kg KBrO_3_, 25 mg/kg indisulam, or 20 mg/kg KBrO_3_ + 25 mg/kg indisulam by intraperitoneal injection once a day for 12 days. Subsequently, subcutaneous xenograft tumours were excised from nude mice, followed by measurement of tumour volume and determination of tumour weight. Tumour volume (mm^3^) was calculated with the formula: *V* = 0.5 × length × width^2^. Finally, tumours were sectioned and stained for HE and IHC analyses.

### 4.16 Organoids Culture

Dissociated HCC21002 and B25013 organoids were resuspended in matrix gel solution, then seeded the mixed solution at 40 μL/well into the 24‐well plate. The plate was placed in the incubator for at least 30 min until the matrix gel was completely solidified, and then 500 μL of pre‐warmed organoid medium (Precedo, Cat. # PRS‐HCM‐3D) slowly added along the wall of each well of the 24‐well plate. Then, the formation of organoids was observed under a microscope after 3 days. When the particle diameter was greater than 50 μm, it was considered that the organoids had formed. The culture medium was replaced every 5 days to continue the culture, and the size of the organoids was kept monitored. Once the majority of organoids were observed to be larger than 50 μm under the microscope and their size no longer increased, the organoids were collected for drug sensitivity tests. Then, organoids were treated with DMSO, 370 μM KBrO_3_, 1 μM indisulam, and 370 μM KBrO_3_ combined with 1 μM indisulam separately. After 144 h of culture, organoids were photographed and the relative inhibition rate was calculated.

### 4.17 Statistical Analysis

The Mann–Whitney *U* test was used for statistical analysis in the Alkaline comet assay, and the error bars indicated the S.E.M. values. Other data were presented as means ± S.D. of at least three biological replicate samples. Statistical and significance analysis were performed using GraphPad Prism 9 and regarded as significant if *p* values were < 0.05. Student's *t* test was used for comparison of two different groups. Significant *p* values are indicated within the figures. *p* < 0.05 (*) is significant, and *p* < 0.01 (**), *p* < 0.001 (***) and *p* < 0.0001 (****) are considered to be highly significant.

## Author Contributions

Z.X., B.S. and H.A. conceived the project and designed experiments. H.A. wrote the manuscript. Z.X. and B.L. supervised the research and critically reviewed the manuscript. Most of the experiments were completed by H.A. B.L. conducted the analysis of TCGA data. A.X. and S.L. conducted the subcutaneous tumour experiment in nude mice. L.G. and D.L. participated in the discussion of this study. All authors read and approved the final manuscript.

## Conflicts of Interest

The authors declare no conflicts of interest.

## Supporting information


**Figure S1.** RBM39 promotes BER in HCC.
**Figure S2.** RBM39 improves cell proliferation and inhibits apoptosis in HCC.
**Figure S3.** RBM39 regulates the expression of OGG1.
**Figure S4.** RBM39 interacts with OGG1 mRNA.
**Figure S5.** Combination of KBrO_3_ and indisulam synergistically inhibits HCC growth.

## Data Availability

The data that support the findings of this study are available from the corresponding author upon reasonable request.
