# Supplementary material for: RBM39 Promotes Base Excision Repair to Facilitate the Progression of HCC by Stabilising OGG1 mRNA
Source: Cell Prolif. 2025 May 13;58(10):e70059. doi: 10.1111/cpr.70059 (PMC12508684; doi:10.1111/cpr.70059)
Supplement: Supplementary file 1 — Figure S1. RBM39 promotes BER in HCC. Figure S2. RBM39 improves cell proliferation and inhibits apoptosis in HCC. Figure S3. RBM39 regulates the expression of OGG1. Figure S4. RBM39 interacts with OGG1 mRNA. Figure S5. Combination of KBrO3 and indisulam synergistically inhibits HCC growth. [file CPR-58-e70059-s001.docx]

**Supplemental Figure**

**
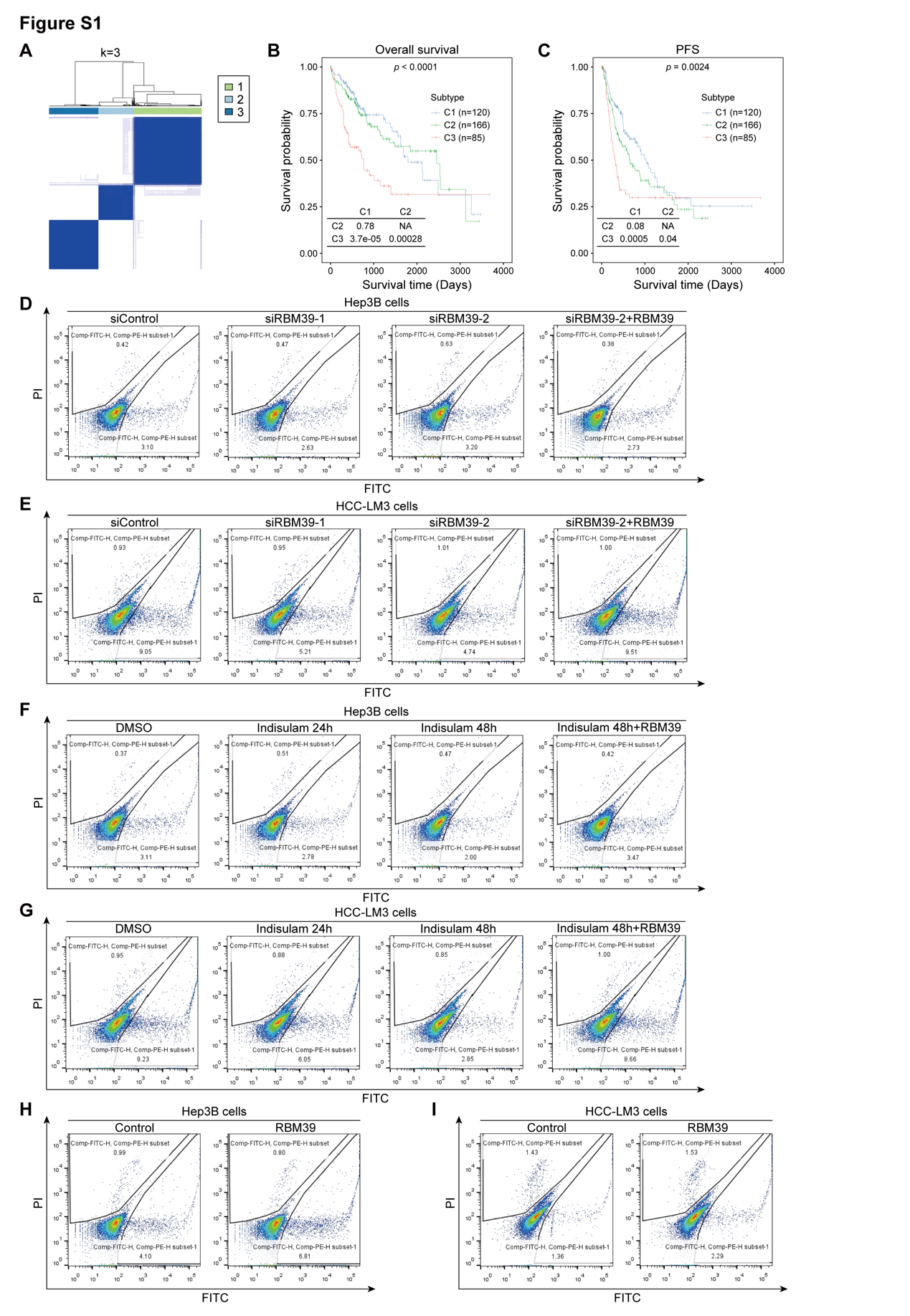
**

**Figure S1. RBM39 promotes BER in HCC.**

**(A)** BER clustering analysis effect for liver hepatocellular carcinoma in TCGA database. Kaplan-Meier curves for overall survival **(B)** and progression-free survival **(C)** stratified by C1, C2, and C3 subtypes in TCGA database. Flow cytometry analysis of BER efficiency in control group and RBM39-depleted Hep3B **(D)** and HCC-LM3 **(E)** cells with transfection of designated siRNAs. Flow cytometry analysis of BER efficiency in Hep3B **(F)** and HCC-LM3 **(G)** cells treated with DMSO or indisulam. Flow cytometry analysis of BER efficiency in control group and RBM39-overexpressed Hep3B **(H)** and HCC-LM3 **(I)** cells. The upper left group represents DsRed^+^ cells and the lower right group represents GFP^+^ cells.

**
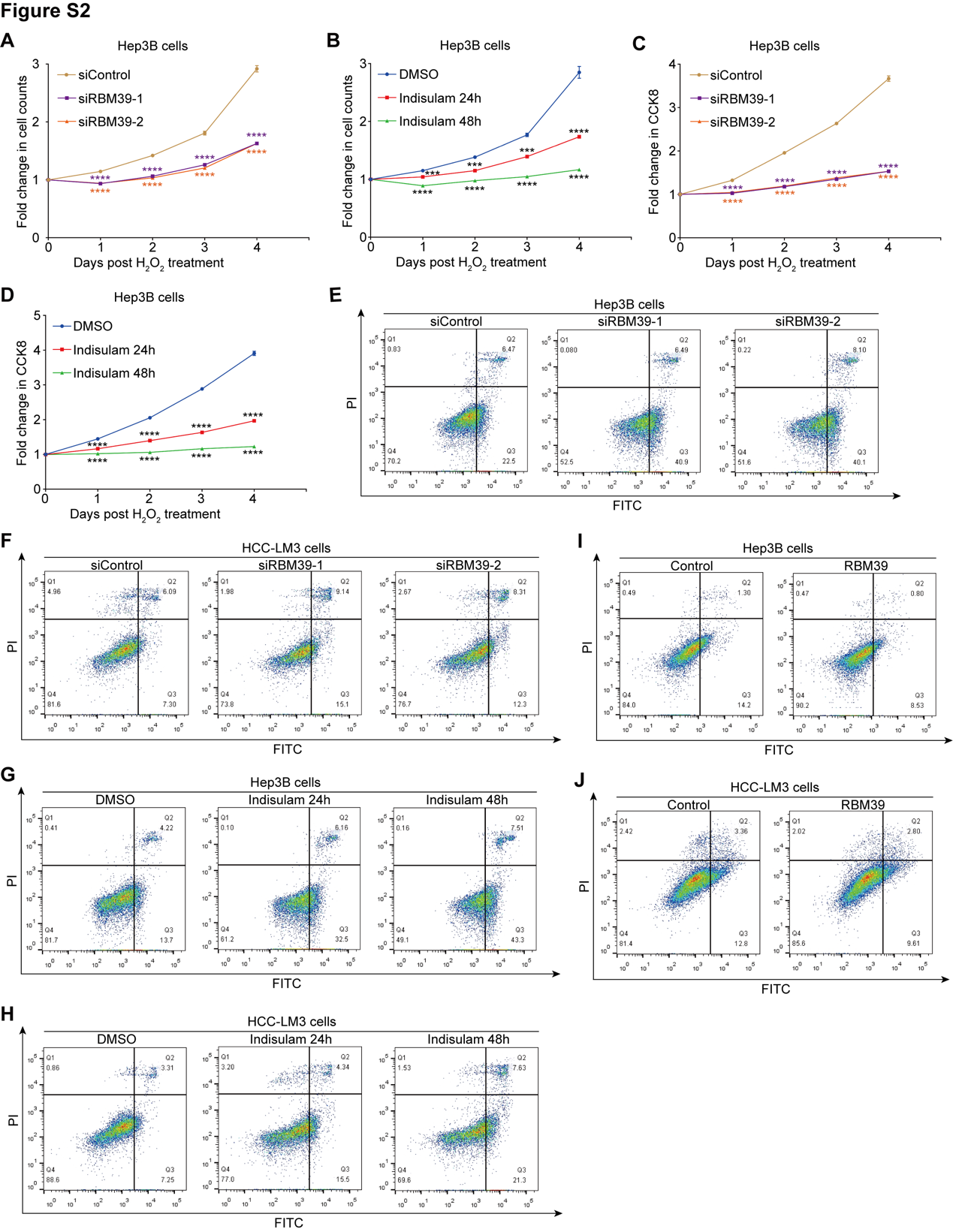
**

**Figure S2. RBM39 improves cell proliferation and inhibits apoptosis in HCC.**

**(A-D)** Cell count and cell proliferation in control group and siRNA transfected- or indisulam-treated Hep3B under the condition of H_2_O_2_. Flow cytometry analysis of cell apoptosis in control group and RBM39-depleted Hep3B **(E)** and HCC-LM3 **(F)** cells with transfection of designated siRNAs. Flow cytometry analysis of apoptosis in Hep3B **(G)** and HCC-LM3 **(H)** cells treated with DMSO or indisulam. Flow cytometry analysis of apoptosis in control group and RBM39-overexpressed Hep3B **(I)** and HCC-LM3 **(J)** cells. The upper right rectangles represent late apoptotic cells, and the lower right rectangles represents early apoptotic cells.


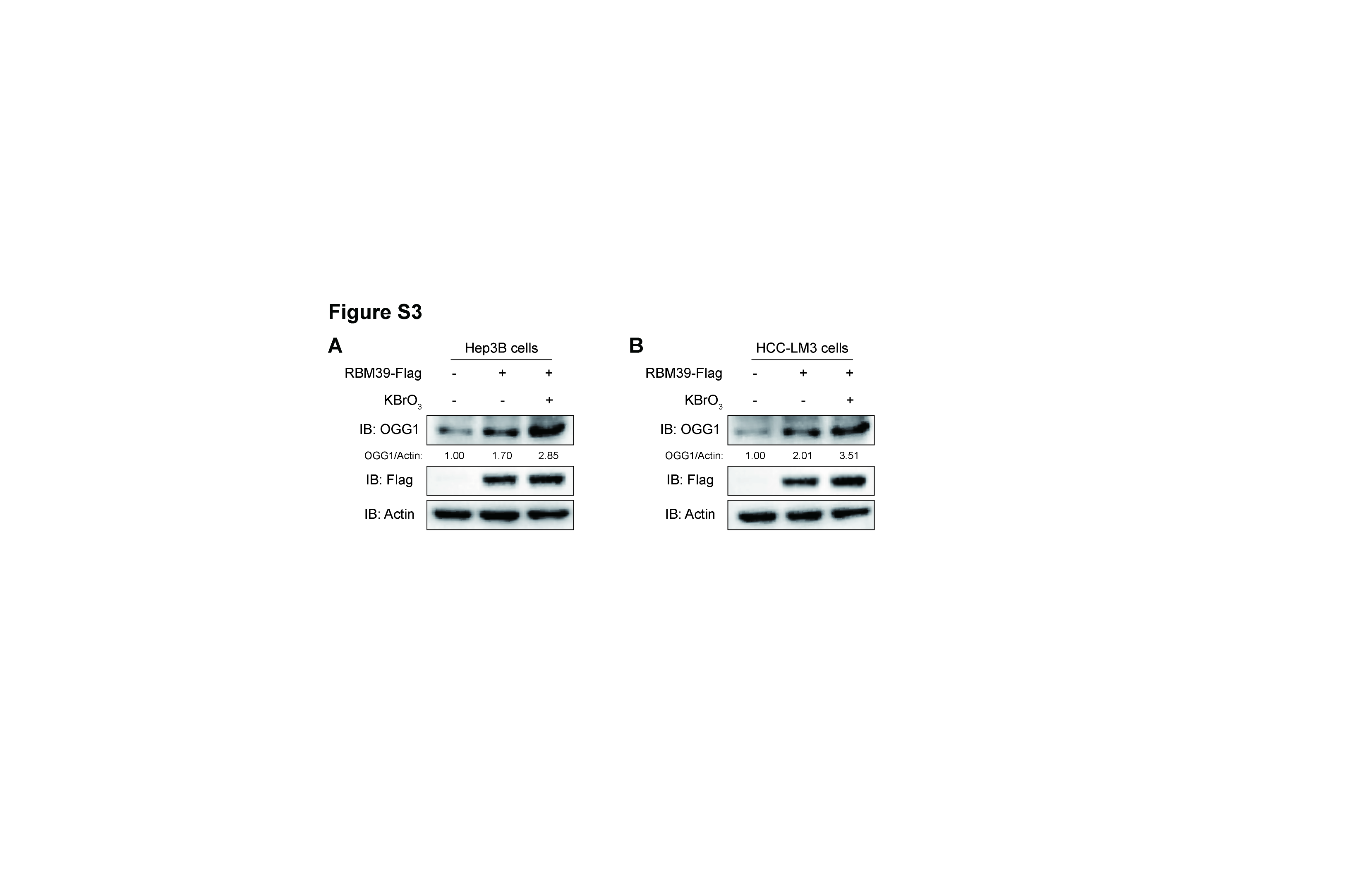


**Figure S3. RBM39 regulates the expression of OGG1.**

The Hep3B **(A)** and HCC-LM3 **(B)** cells transfected with RBM39-Flag were treated with KBrO_3_ at 40 mM for one hour. Samples were collected for western blot analysis to detect the OGG1 protein levels.


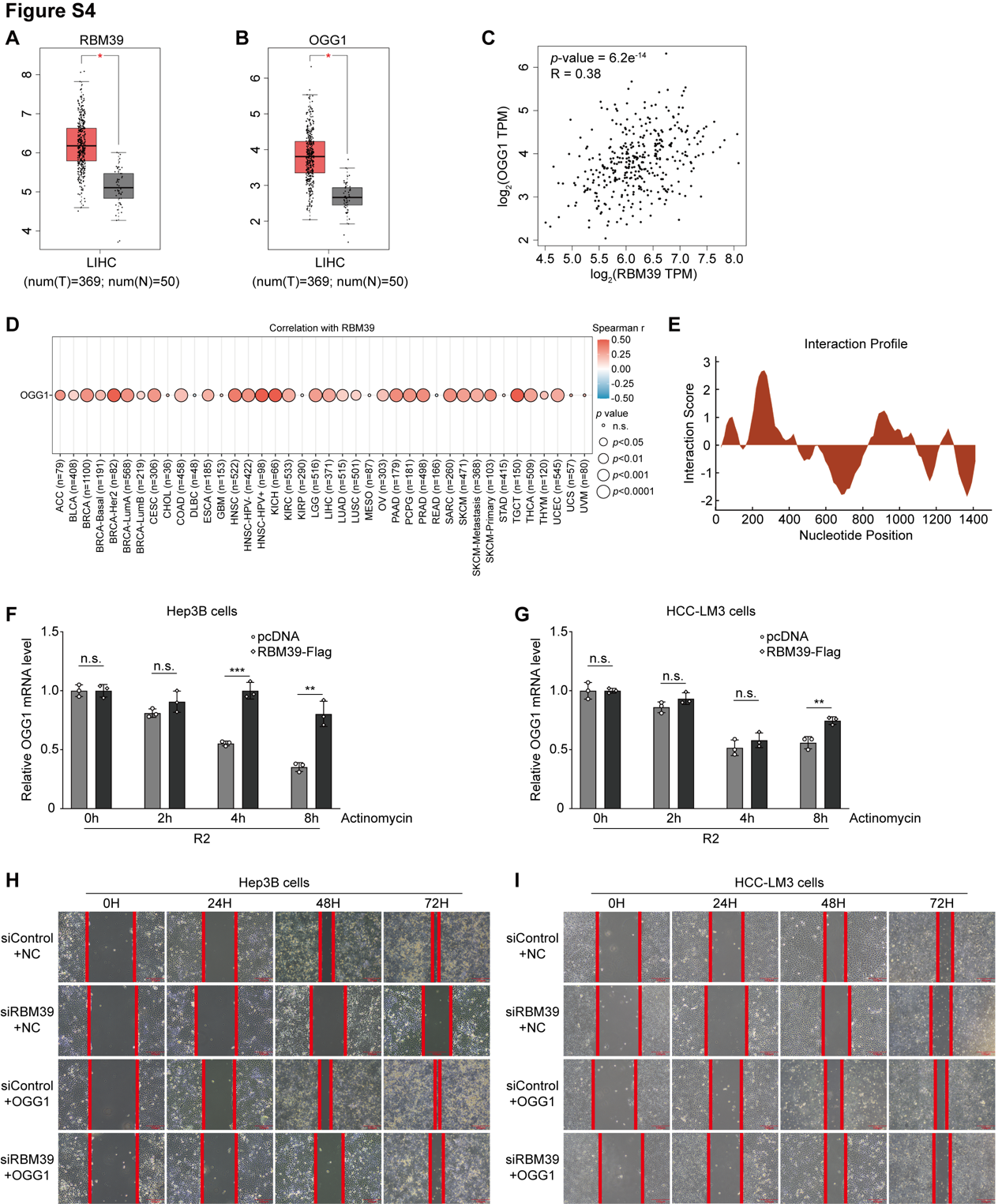


**Figure S4. RBM39 interacts with OGG1 mRNA.**

Analysis results of RBM39 **(A)** and OGG1 **(B)** expression levels in human liver tumor tissues (T; n = 369) and adjacent nontumor tissues (N; n = 50) in the TCGA database. * P < 0.05 . **(C)** The correlation analysis of RBM39 and OGG1 in human liver hepatocellular carcinoma in the TCGA database. Spearman correlation coefficient was used for analysis. **(D)** The correlation analysis of RBM39 and OGG1 in multiple human cancers in the TCGA database. **(E)** The potential binding regions between RBM39 and OGG1 mRNA by the catRAPID database (<http://service.tartaglialab.com/page/catrapid_group>). The Hep3B **(F)** and HCC-LM3 **(G)** cells in control group and RBM39-overexpressed group were treated with actinomycin at 5 μg/ml for the indicated times. Samples were collected for quantitative RT-PCR analysis to detect the expression level of OGG1 using R2-F2 primers. Overexpression of OGG1 rescued the wound closure of Hep3B **(H)** and HCC-LM3 **(I)** cells with RBM39 knockdown.** *P* < 0.01; *** *P* < 0.001; n.s., not significant.


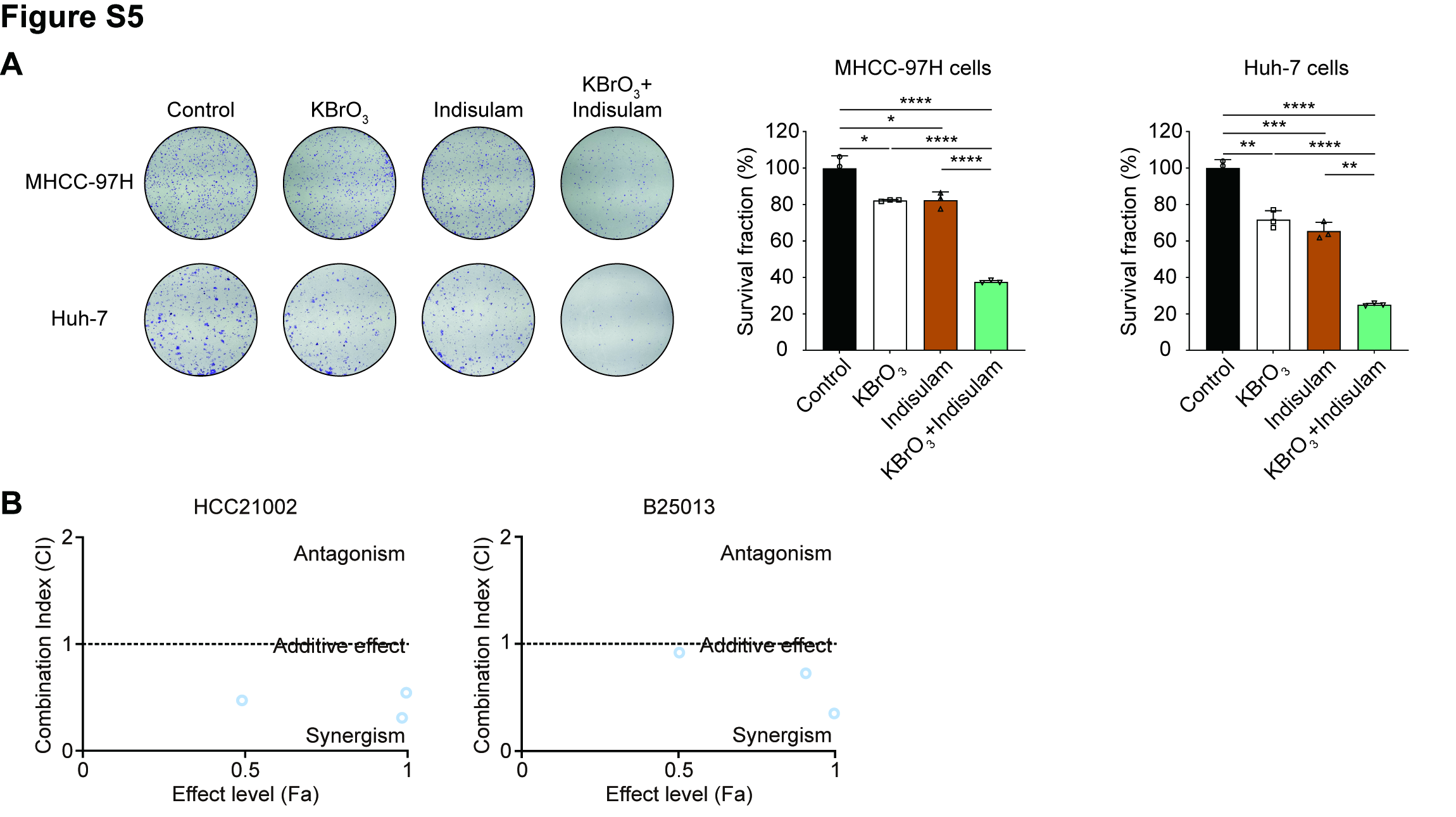


**Figure S5. Combination of KBrO_3_ and indisulam synergistically inhibits HCC growth.**

**(A)** Colony formation images and colony count results of the indicated-treated MHCC-97H and Huh-7 cells treated with DMSO, 20 mM KBrO_3_, 1 μM indisulam or 20 mM KBrO_3_ combined with 1 μM indisulam. **(B)** The Combination index analysis of the combination treatment of KBrO_3_ and indisulam in HCC21002 and B25013 organoids. A combination index <1.0 indicates a synergistic effect. * *P* < 0.05; ** *P* < 0.01; *** *P* < 0.001; **** *P* < 0.0001.
